# Difficulties in Caring for the Older Adults: Perspective of Brazilian and Portuguese Caregivers

**DOI:** 10.3390/nursrep13010027

**Published:** 2023-02-16

**Authors:** Elaine Santana, Felismina Mendes, Joana Bernardo, Rosa Silva, Pedro Melo, Pollyanna Lima, Alessandra Oliveira, Luciana Reis

**Affiliations:** 1 Health Sciences Research Unit, Nursing, Nursing School of Coimbra (ESEnfC), 3000-232 Coimbra, Portugal; 2 S. João de Deus School of Nursing, University of Évora, 7000-811 Évora, Portugal; 3 Center for Health Technology and Services Research (CINTESIS), Nursing School of Porto, 4200-450 Porto, Portugal; 4 Portugal Centre for Evidence Based Practice, A JBI Centre of Excellence (PCEBP), 3000-232 Coimbra, Portugal; 5 Institute of Health Sciences, School of Nursing (Porto), Centre for Interdisciplinary Research in Health, Universidade Católica Portuguesa, 4200-450 Porto, Portugal; 6 Independent School of the Northeast, Collegiate of Nursing, Vitória da Conquista 4555-030, Brazil; 7 Health Departament I, State University of Southwestern Bahia, Jequié 45205-490, Brazil

**Keywords:** caregivers, aged, functional status, caregiver burden

## Abstract

This study aims to understand the difficulties in caring for the older adults with functional dependence from the perspective of Brazilian and Portuguese caregivers. This is a study based on the Theory of Social Representations, based on the Thematic Content Analysis proposed by Bardin, carried out with 21 informal caregivers of older adults in Brazil and 11 informal caregivers of older adults in Portugal. The instrument consisted of a questionnaire with sociodemographic data and data on health conditions along with an open interview with guiding questions on the theme of care. Data were analyzed using the Content Analysis technique proposed by Bardin, with the help of the QRS NVivo^®^ Version 11 software (QSR International, Burlington, MA, USA). Three categories emerged from the speeches: “Caregiver burden”, “Caregiver support network” and “Older adults resistance”. The main difficulties mentioned by caregivers were associated with family articulation in meeting the needs of their older adults, whether due to the excessive demand of tasks, which results in overloading the caregiver, or the behaviors of the older adults themselves, or even the availability of a truly supportive and effective network.

## 1. Introduction

The aging of the population is a concrete reality, which in recent years has been widely discussed [1,2]. Several facets of this phenomenon deserve to be highlighted and, with regard to the need for care that comes with age, informal caregivers and the family scenario emerge as a response to needs, but also as an issue, that deserves special attention.

With advancing age and limiting health conditions, it is common for a significant number of older adults to live with high levels of functional dependence and because of this they need help from others to carry out basic daily activities of life, such as hygiene, food and walking [3,4,5]. It is in this sense that the family begins to play the role of the main caregiving institution, often needing to form a new family arrangement to adapt to the new reality [6,7,8], and experiencing conditions of work overload and family conflicts [2,9,10].

Being a family caregiver is an arduous task, pointed out by the literature as a complex function that requires not only mastery in the performance of tasks, but also common sense and emotional control [11,12].

The complexity of care may vary according to the learning capacity that caregivers have in relation to routines and tasks, as well as the interaction with older adults in order to encourage their participation in the process. Furthermore, it is only through experience as a caregiver that family members are able to truly understand the meaning of such a role, recognizing their personal limits as they are constantly immersed in a process of self-assessment [10,11,13].

Faced with the specific dependency and the imposed care demand, family members, who become informal caregivers seek to organize themselves in the best way to provide care, but there are two factors to be considered at this time. The first concerns the previous preparation of caregivers for the development of the function and the second is related to the practical conditions of the same.

With regard to preparation to exercise the role of caregiver, what family members have reported is that there is no notice, or no prior moment for the family to organize itself and assume such responsibility, or even consider it as an option. From the diagnosis, it is necessary to consider elements such as the evolution of the disease, the conditions and skills of the family members, the existence of a family network, the place that the chosen caregiver occupies in the family, as well as the time and effort available, in an attempt to adequately manage the changes and the needs [2,10,14].

Regarding the practical development of care, the findings in the literature demonstrate that, in most cases, carrying out daily activities and special care cannot be performed without compromising the caregiver’s quality of life [1,15,16,17]. Studies published in this area confirm that care provided by family members in the home environment can cause damage to the informal caregiver, whether at a physical, psychological and emotional level, or at a social level, characterizing care as the background to understanding the illness process of these individuals [14,18].

The main elements that hinder the performance and adaptation of caregivers to care are related to the caregiver’s age, emotional and physical health conditions, personal life activities, work activities, financial burden and insufficient family support [14,19,20].

The functional dependence of the older adult relative can cause damage to the caregiver’s life, and there may be five types of crisis: (i) awareness of dependency, in which the caregiver feels powerless in the face of the disease; (ii) unpredictability, associated with the perception of disease progression as an inherent condition of aging; (iii) time constraints, related to the progression of dependency and therefore the need for more intense care, causing, in turn, greater wear and tear; (iv) relationship between peers, which concerns possible conflicts arising from the reversal of roles and the relationship prior to dependence; and finally, (v) the lack of choice, which is closely related to the fact that the caregiver is elected due to the absence of other individuals to exercise the function [18].

In this sense, the importance of investigations of this nature must be highlighted, which seek to enter the home environment and reveal the reality experienced by caregivers and by the older adults themselves. The unveiling of conditions that involve care, especially in the domestic environment, is a notable strategy, both for allowing the recognition of the situation in a factual manner and for favoring the perception and elaboration of social and political responses through such findings.

Considering the social nature and the historical, moral and cultural issues that surround this phenomenon, the theory of social representations (TRS) was used to explore the issue from a broad and multidisciplinary perspective [21].

TSR represents a form of socially constructed practical knowledge whose function is to give meaning to the reality of everyday life. As a particular way of acquiring knowledge and communicating it, social representations seek to order the knowledge acquired from the perceptions that are produced in the world [21].

Essentially, TSR enables individuals not only to experience a reality, but to appropriate it and internalize its social concept, attributing meanings to their reality from this relationship and, consequently, building social representations of a given object by means of social interaction [21,22].

Considering that all social representations have the function of making something unfamiliar familiar [21], it is through them that members of a community can establish communication. The SRT provides the codes and symbols by which individuals classify and identify the elements and phenomena of their daily social and individual life [23].

The present study aims to analyze the difficulties in caring for older adults with functional dependence from the perspective of Brazilian and Portuguese informal caregivers.

## 2. Materials and Methods

This is an exploratory, descriptive and qualitative study, based on the Theory of Social Representations [21], which was part of the project *Qualification of caregivers and aspects related to the quality of life of dependent older adults people in primary and tertiary care: proposal, implementation and evaluation of protocol* developed at the State University of Southwest Bahia, Brazil, and at the São João de Deus School of Nursing at the University of Évora, with the participation of informal caregivers of older adults registered in assistance programs at the Municipal Home-Based Care Program for Older People with Disabilities (Programa de Atendimento Municipal Domiciliar ao Idoso com Limitação (PAMDIL) in Vitória da Conquista, Bahia, Brazil, and the Integrated Community Care Team (ECCI) in Évora, in the Alentejo region, Portugal.

The sample was defined by convenience, as a result of a graduate exchange (PhD sandwich) carried out by the first author.

PAMDIL is registered in the third largest city in the state of Bahia, with about 306,866 inhabitants, corresponding to the health hub of the southwest region of the state [24]. This program, in operation since 2009, assists around 1000 seniors, identified by community health agents, with some type of limitation. The older persons receive fortnightly visits from the team, consisting of two doctors and two nursing technicians. In addition to consultations and home care, which can be anticipated as needed, the older person, if needed, can receive referrals to other medical specialties.

The city of Évora, which hosts the ECCI in question, is also the capital of the district of Évora (made up of 14 municipalities) with a population of 53,084 inhabitants. The Alentejo is considered the most aged region in Portugal and the proportion of older adults per 100 young people in the city of Évora is 161.6 [25]. With regard to the program, the ECCI operates as a full-time monitoring service for users, most of whom are older people. The integral support provided by ECCI works with face-to-face assistance during 12 h a day and by telephone during the other 12 h and on weekends.

The participants of this study were informal caregivers of older adults with functional dependence from Brazil and Portugal, these being 21 Brazilian informal caregivers and 11 Portuguese informal caregivers. Caregivers were identified from the records of the two programs. Together with the Community Health Agents in Brazil, and with the members of the ECCI team in Portugal, home visits were made to the identified addresses to formalize the invitation to participate in the study.

In the Brazilian scenario, 176 home visits were carried out, however, n = 23 were excluded due to the death of the elderly person and n = 52 due to refusing to participate in the study or no longer residing at the indicated address, thus totaling 101 effective homes, which after applying the criteria inclusion criteria resulted in 21 participants.

The inclusion of informal caregivers was based on the following criteria: having some degree of kinship with the older person, residing in the same household, being over 18 years of age, of any gender or marital status, and being the main responsible person for the care of the older person (without remuneration).

The research instrument consisted of two parts: the first consisted of a questionnaire with sociodemographic data and health issues such as gender, age group, education, marital status, profession, family ties and health problems. The second part was then constituted by the application of an open interview with guiding questions.

Content Analysis was used to analyze the collected information, proposed by Laurence Bardin with the help of the QSR NVivo^®^ software, version 11. Content Analysis consists of a set of communication analysis techniques and, according to Bardin [26], through this technique it is possible to deal with the information contained in the messages and thus achieve an exploration of both the meanings and the signifiers.

Regarding the QSR NVivo^®^, it is a software that helps in the organization and structuring of data. Through the organization of the information collected through the interviews, NVivo enables a kind of categorization in which the descriptive information of the text is arranged based on the identification of trends and, through this structuring, “nodes” and “sub nodes” are created and, in a second step, word clouds are also created with the words most frequently cited by the participants. This feature allows for better visualization of categories, in addition to representing a modern device that contributes to content analysis [27].

Both studies were submitted and approved by the Research Ethics Committees. The Brazilian Project was approved by the Research Ethics Committee of the State University of Southwest Bahia under number 1,875,418 on 15 August 2016 and the Portuguese project was approved by the Ethics Committee of the Health and Well-Being Area of University of Évora with the number 16,012 on 19 May 2016.

## 3. Results

**Figure 1:**
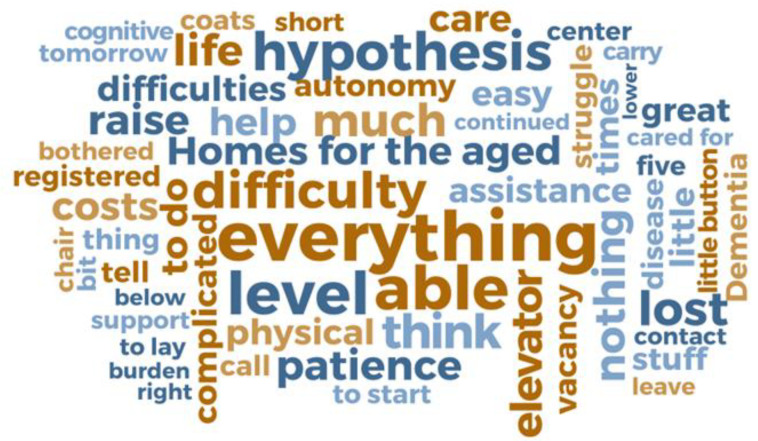
Word cloud. Difficulties in carrying out care by Portuguese caregivers. Via QSR NVivo^®^.

**Figure 2:**
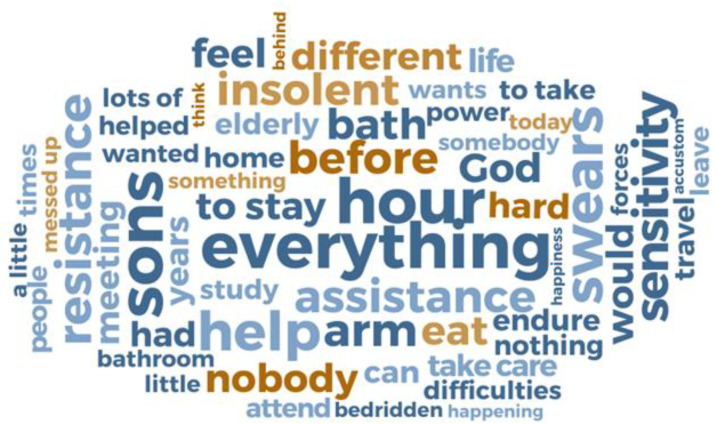
Word cloud. Difficulties in caring pointed out by Brazilian caregivers. Via QSR NVivo^®^.

The word clouds (Figure 1 and Figure 2) illustrate the main difficulties reported by caregivers, highlighting mainly work overload, lack of support and resistance of the older adults to care.

In the word cloud of Portuguese caregivers (PC) (Figure 1) the words “everything”, “capable”, “complicated”, “bandage”, “capable” and “elevator” confirm that the routine of these individuals consists of carrying out many activities, because due to the installed functional dependency the older adults are no longer able to perform some basic life activities alone and for this reason they require the caregiver at various times of the day.

In the word cloud of Brazilian caregivers (BC) (Figure 2), the similarity revealed by the evocation of Portuguese caregivers through the element “everything” and other terms such as “help”, “sons”, “swear”, “resistance” and “difficult”, indicate the centrality of difficulties in the lack of help from other sons and the older person’s own resistance to care.

Based on the analysis of word clouds and interview excerpts, three categories were created: “Caregiver burden”, “Caregiver support network” and “Older adults’ resistance”. Next, the developed analytical categories are presented.

### 3.1. Caregiver Burden

In the category “caregiver burden”, social representations of the difficulties of the care process unveiled by caregivers of Brazilian and Portuguese older adults are presented.

When questioned about the difficulties encountered in carrying out their role, the participants indicate the wide list of activities they need to perform daily, mention the physical effort required of them and even refer to the lack of freedom with which they find themselves. Participants in both groups recognize that overwork is a complicating factor, which can be confirmed in the fragments presented below:

As she is totally dependent, due to urinary incontinence, she doesn’t have the strength in her legs for me to hold her, for example, to take her out of the chair and sit on the toilet. Getting her out of the toilet and sitting on the chair has to be all push. This is too hard. And then take her out of the chair to put her in the other chair to go to the bathroom, that you have to take off diapers and you have to protect the chairs, a person alone has a lot of difficulty… holding her well with you, but then this effort affects me a lot because I suffer from my back. PC_3.

I bathe him, make him eat, wash, dress him, bring him out here to eat, to walk a little, if he goes to the bathroom I have to go with him to hold his arm, I’m afraid of let it fall. He already fell in that bathroom which is so small, if he falls and hits his head on the toilet or on the basin, or on the shower, he can kill himself. But the bath is very expensive, but he doesn’t want help. He says: I don’t want Caritas here, my Maria is the one who gives me the bath. PC_11.

I don’t have that much freedom. I can’t go out with my friends and arrive too late. I have to get back early, I can’t sleep. Because I have to take care of her. I go out, but my mind is on her… But I have to give up more of myself, to be able to take care of her. I think about her more than myself. BC_06.

In addition to the aforementioned work overload, the caregivers’ speeches suggest an implicit condition, associated with the existence or insufficiency of a support network.

### 3.2. Caregiver Support Network

In the category “Caregiver support network” we find how this element, despite different behaviors reported in the two scenarios, is referred to by the participants as a difficulty.

Portuguese caregivers initially describe the formal support network as a support resource, as can be seen in the excerpts described:

Now I don’t bathe them anymore because they bathe them twice a week. When they came I was really down, half of what I am here. I already got better. He goes at eight and comes back at seven. And now he sleeps because of the pills they give him. Yesterday he stayed at home because it was Sunday, right? PC_1.

My husband was in a day care center for five months, but now he got bored with it and this month he didn’t want to go. Now he’s home. I put his name to another, because maybe changing places… but he said he won’t go anywhere else. And my father, during the week, Caritas goes there to bathe him, dress him, and I only change the diaper during the day and night. Lay him down, undress him, put on his pajamas and change him. PC_4.

However, these mentioned services do not sufficiently meet the needs of an increasingly aging and dependent population and for this reason Portuguese caregivers, following the questioning, endorse the difficulties they encounter regarding this support network:

Because those services are like that, medium-term continuous care. The day she enters, she already knows the day she will be discharged. I’m going to the meeting with the Doctor and see if they can grant home care. I’ll take care of it, because there’s no other option. She was retired very early on account of her disability and doesn’t have a decent retirement. I’m going to call home support now and I don’t even know how it’s going to be. I have her registered in the homes for the aged. It is a hypothesis, in the short term, because vacancies in nursing homes cannot be requested in advance, we cannot say “let’s kill the one who is older”, we have to wait for someone to leave. This is how it is, or a transfer or someone who dies and then as soon as the vacancy occurs there is no other way and we cannot predict this either. Continuous care, when there is a vacancy, they call, but they cannot say it is today or tomorrow, it is only when they have a vacancy. PC_3.

One of the issues, especially in relation to dementia, is the lack of specialized support. Because, for example, in the said homes, there are none close by. There is in Lisbon. At the local level the offer is very limited and there are general homes and, therefore, I think it is more complicated there. It’s one thing when I know what’s happening, what’s going to happen, how can I intervene, how can I not intervene, and another thing is when all of a sudden… but I think that support is much more important initially, when you start to understand what is happening, it has an impact on her, on the caregiver, on the children. PC_7.

References to support from Portuguese caregivers were only related to formal support, which allows the inferring of a satisfactory assessment of the informal network, in addition to reflecting how the care action has been focused on a mutual participation between the State and the family, with the aim of reducing the caregiver burden established in the informal sector.

On the other hand, the references of Brazilian caregivers, which will be presented, denote the differing reality experienced when comparing the two countries. The speeches evoked by these participants are centered on the difficulty encountered in the face of the lack of support, not only in the formal network, but mainly due to the deficient informal support that results in an obvious burden to the caregiver.

Not now that her son arrived from São Paulo. And before it was more difficult because I had to keep company and I couldn’t go to study. I decided to stop (from studying) to take care of her. So much so that even to be able to go to school at night, I already leave dinner ready, I already leave her showered, I already leave everything. BC_3.

Here’s the thing, we have to ask God for strength. What most upsets us is knowing that people also have an obligation and do not fulfill it, then we are forced to fend for ourselves. It’s difficult. Unless justice really helps. BC_9.

Messed up everything. My life stopped in time. It could be different if the children helped me with them. Things were happening little by little and then they found support, so let’s lean on it. Eight children is no joke… BC_13.

I ask God for strength. Because I take care of her alone, nobody helps me with anything, but there are more children, but only I take care of them. I see the other daughters treating her with neglect, they don’t give her affection or attention. BC_17.

Family caregivers also mention that, in the process of caring for the older adults, resistance tends to represent a complicating factor.

### 3.3. Resistance of the Older Adults

In this category, representations are presented that demonstrate how the profile of the older person, or the way they deal with the condition of functional dependence, influence the care process and the caregivers’ perception of the phenomenon. This can be seen in the following excerpts:

The way he is, you can’t talk to him much. It doesn’t work because his character doesn’t do much for that. Because whatever is said against his ideas, he is not an accessible person and he never was, to tell you the truth, not after he was sick, he never was… he was always a bit aggressive… PC_8.

What I feel the most is that she says very little. Able to spend hours without saying anything and that’s what I find strange the most. It has changed a bit, this is the difficulty, she doesn’t like to do anything, it costs me a lot. Life was a normal life, and from then on things changed. PC_9.

He is very stubborn. That you have to eat, that you have to go out for a bit to sunbathe, that you need to take a shower. These difficulties I encounter. The resistance he has. I wish he had more of the joy he had before. BC_7.

He mistreats in words. Sometimes I find words hard. Here at home, if he doesn’t talk, attacking us with words, there’s no talking here […] I don’t even try to talk to him. We don’t look for a fight with him, but he… […] If you say “let’s do physical therapy?” he “I’m about to die” can you oblige? I can’t. I can’t make him go to the doctor. He and I hardly talk much. Because he swears at me a lot. He’s very stubborn, he’s very bold. He calls us a lot of ugly names. He’s not bad, but he’s cheeky. When he wants to talk, he says what he wants with us! So now he curses me a lot, but I don’t answer him much. I prefer to remain silent. BC_14.

## 4. Discussion

According to TRS, social representations allow shaping and restoring collective consciousness, as they explain events and objects in such a way that they become accessible to any individual [21].

To be created, social representations encompass two interconnected mechanisms: anchoring and objectification. Anchoring consists of the assimilation, the classification we make of a certain object/phenomenon based on the value systems that this object has in the social environment in which we are inserted. Objectification, on the other hand, comprises the materialization of ideas, in the reproduction of a concept in an image [21]. In this way, the social representations revealed by the two groups of caregivers symbolize and reproduce the senses and meanings attributed to the difficulties encountered in the performance of their function.

Burden was a point strongly mentioned by both groups of caregivers. As it was possible to observe in the word clouds, the evocation of the element “everything” reinforces the existence of a work overload in the routine of these caregivers.

The term “burden” is commonly replaced by alternative terms such as stress, tension or anguish, and corresponds to the set of physical, socioeconomic and psychological problems that can affect the caregiver’s life in social, family, freedom and work relationships, and emotional balance [13]. It constitutes a resistance to the provision of care due to the high demand of tasks performed, which are influenced, for example, by the degree of dependence of the older person or the offer of help available to caregivers [28].

The discussion around this theme has been approached with great frequency in research, as the number of caregivers who develop health problems as a result of exercising the role of caregiver is increasing.

As noted in the participants’ statements, what can also be seen in the literature is the great effort involved in carrying out daily care, which tends to increase as the difficulties and dependency of the older person progress [29]. Such conditions are recognized by the caregivers, who composed the sample of this work, and confirmed in other studies [16,17,30] pointing out how the demand for care overloads their lives and causes illness, quite common in this practice. They direct their attention completely to the older adults, forgetting themselves and neglecting their own interests, hobbies and even health [5].

When reporting that in providing care they are responsible for a wide range of decisions, attributing to the element “everything” the value of the commitment they have in the life of the older adults, caregivers demonstrate once again the work overload that has been reserved for them and how this is an intense and full-time experience.

In this context, what the studies point out is how the time dedicated to the exercise of care is related to higher levels of overload and, consequently, greater suffering among informal caregivers [2,18,31,32].

The negative feelings relate to concerns regarding the progression of the older person’s disease, or with questions related to their own health. The stress resulting from the intense routine, possible family conflicts and uncertainty in future issues, as well as the anguish due to the renunciation of their personal life, are some of the many factors that affect the lives of caregivers and that affect both the performance of care and their well-being and quality of life [2,28].

In addition to the determining factors for the overload of informal caregivers mentioned above, the support network, which characterizes the second category (caregiver support network), is also referred to in the literature as an element of significant influence in this process [2,10,15].

Studies developed on this topic, which sought to assess the relationship between the support network and the overload of informal caregivers, show that, when caregivers can count on greater quality social support, they tend to present lower levels of burden [2,28].

Support networks function as an important resource in helping older adults and their family, whether in providing health care, with household chores or even financial and emotional issues. These networks can be characterized as formal or informal [33,34]. The informal support network is the one linked to family, friends, neighbors and social groups such as churches. The formal network consists of offering support through formal organizations available through public policies such as health services, hospitals, long-stay institutions and government programs with the assistance of professionals in nursing, medicine, psychology and social work [33].

An important consideration of this aspect is how the discourses of the Portuguese and Brazilian participants are similar when they evoke issues related to the respective support networks, but differ in the references made to each of them.

The speeches of the participants that emerge from this category portray the cultural and practical inequalities, the advances and the different social responses that exist between the two countries. Beforehand, two central issues guide this approach and are based on offering a formal support network for Portuguese caregivers, while in the Brazilian reality, considering that this type of device is still quite scarce, which is reflected in the caregivers’ speeches, there is protest about the presence/absence of the informal network.

What has been observed in the Portuguese reality is that, even in the face of changes in the structure of households and the historical-cultural trend towards institutionalization, Portuguese families, in correspondence with other countries in Southern Europe, have increasingly adopted a strategy for coping with old age mixed care profiles [35,36,37]. These mixed profiles consist of providing care based on the use of two types of support available: the family, primarily responsible for the informal support network which has experienced a high level of overload, and the formal network which, despite the reduced offer, has expanded in view of the reforms and the need to expand access [38].

Social responses are provided by initiatives such as Casas de Misericórdia and Private institutions of social solidarity through the Home Support Service, Day Centre, Night Centre, Community Centre, Family Shelter for older adults and Residential Structure for older adults [25]. In 2006, complementing the network, the National Network of Integrated Continuous Care was created, guaranteeing health care and social support for older people through different units (convalescence, medium, long-term and palliative) to be carried out by the Teams Integrated Continuing Care [25].

The provision of care facilitated by the formal network can be divided into health care, which is a type of free service for the patient and financed exclusively by the Ministry of Health, and social support, which can be paid for by the patient or shared with Social Security, depending on the financial conditions and income of the older person and family [32]. In addition, in 1989 a subsidy was created for informal caregivers called the Supplement for dependency, but due to constant reforms in assistance policies, one measure was implemented and started, excluding individuals with incomes greater than 600 euros per month from the benefit [5,39].

However, the complaints reported by Portuguese informal caregivers show the lack of response from these devices, which contributes to dissatisfaction with the formal support network, and consequently to the overload of these caregivers. In the literature, it appears that the need not met by formal services is revealed as a significant element for the occurrence of caregiver burden [2,15,40].

With regard to the Brazilian reality, it is common that, in family reorganization, the provision of care is attributed to a single member, elected as the main caregiver, on whom the greatest share of responsibility for the older adults will fall [41].

The implications of this accountability end up reflecting the negative consequences in this process. Studies show that feelings of loneliness, and exhaustion, are evidenced, mainly, among the main caregivers who do not find in other family members the necessary support to dedicate themselves to the care of the older adults, but also to other interests in their personal life [18,42,43]. In addition, other issues associated with the intense dedication required in care are revealed in the financial sphere, since informal caregivers are often unable to develop paid work outside the home [44].

Studies developed within the scope of family functionality demonstrate the relationship between higher levels of burden and lower levels of family functionality [44,45,46,47].

In this sense, satisfaction with family functioning and, consequently, the help coming from other family members or members of the informal network represents an aspect of great value to the main caregiver, since in this way the demands and the workload are attenuated, improving their perception of health and the pronounced changes that have occurred in their lifestyle [5,19,44,48,49].

Regarding the perception of resistance on the part of the older adults revealed by informal caregivers in the third category, the statements demonstrate how the temperament of the older adults, and how they position themselves in the face of dependence, have a great influence on coexistence and routine care.

This assertion was also reported in other studies that indicated how the behaviors of older adults, namely mood swings, intergenerational conflicts, the difficulty of the older person in recognizing people or places and their own resistance to care, were referred to as difficulties by caregivers [41,50,51,52].

It is natural that, faced with a complex and subjective experience such as caring, and the circumstantial imposition which commonly determines who will assume such a role, caregivers refer to the older person’s attitudes as a facilitating or aggravating element of the process. Considering that most caregivers are family members, namely spouses and daughters, the memories and family relationships built throughout life exert a strong influence on the way they deal with this reality.

Studies developed with female caregivers of older adults highlighted this aspect as something relevant in the care process [47,53]. Similar to the results found in this study, in the discourse of the women who provided care for older husbands, it was possible to perceive that references to husbands, with behaviors and characteristics of stubbornness, aggressiveness and temper, represent a reflection of the costs to the daily life of those who take care.

On the other hand, given the range of clinical conditions that can culminate in the establishment of functional dependence, informal caregivers may refer to difficulties in exercising their role as characteristics of the condition that led the older person to need care. As mentioned by the participants in this study, mood swings, the older adults’ resistance to care and the situation of dependence, as well as difficulties in accepting the family member as a caregiver, were characteristics revealed in other studies with caregivers of older adult victims of stroke and older people with dementia and Alzheimer’s disease [50,54,55,56,57].

The approach referred to in this topic, added to the other aggravating factors presented above, not only justifies the specific aspect of, and the urgency of, responses to the needs of caregivers and older adults, but also denounces significant aspects that compromise the balance, functioning and quality of care offered.

Social representations of the difficulties in caring for older adults revealed by the participants of this study allow not only the uncovering of important questions about the impairment of the quality of life of informal caregivers, but also demonstrate how this phenomenon is surrounded by complexity and cultural and historical moral influences. Despite the constraints and challenges encountered in carrying out this function, moral and historical aspects such as family ties of marriage and parenthood, or even the very social construction of residential institutions for the elderly, act as determining factors in the choice/obligation to care [49].

One of the limitations of this study was the sample size which, despite demonstrating through social representations a reality that has been proven by other studies, had a small number of participants.

Future studies should include the participation of informal and formal caregivers, from different professional categories, and also of older adults, in order to raise the point of view of the actors integrated into the formal care network, promoting the discussion of this theme from the perspective of the greater number of people involved in the care system.

## 5. Conclusions

The responsibility of caring for older adults at home imposes on informal caregivers an intense and often full-time work routine. The main difficulties mentioned by caregivers were mainly associated with the increased demand of the tasks and responsibilities required by care, which results in burden. Another factor pointed out by the participants denotes the difficulty in family articulation in meeting the needs of their older adults, whether in reference to the behavior of the older adults themselves, or in the availability of a support network that is truly effective.

What draws attention to this issue is the divergence in the experiences of each of the groups. While for Portuguese caregivers, the difficulties reported are correlated with the inconveniences of accessing the formal support network, Brazilian caregivers reveal the fragility of this sector in the country, as they do not even mention it, contesting, mainly, the difficulty in gaining the support of the informal support network.

Discussions like these are fundamental in understanding care and constitute a very important tool referred to in the gerontological literature, in the sense of revealing the anguish and difficulties of informal caregivers and allowing effective responses to be thought through and elaborated according to their needs.

## Data Availability

The datasets of the current study are available from the corresponding author on reasonable request, contactable via elainesantana@esenfc.pt.
